# HLA Association with Drug-Induced Adverse Reactions

**DOI:** 10.1155/2017/3186328

**Published:** 2017-11-23

**Authors:** Wen-Lang Fan, Meng-Shin Shiao, Rosaline Chung-Yee Hui, Shih-Chi Su, Chuang-Wei Wang, Ya-Ching Chang, Wen-Hung Chung

**Affiliations:** ^1^Whole-Genome Research Core Laboratory of Human Diseases, Chang Gung Memorial Hospital, Keelung, Taiwan; ^2^Department of Dermatology, Drug Hypersensitivity Clinical and Research Center, Chang Gung Memorial Hospital, Linkou, Taipei, Taiwan; ^3^Research Center, Faculty of Medicine Ramathibodi Hospital, Mahidol University, Bangkok, Thailand; ^4^Chang Gung Immunology Consortium, Chang Gung Memorial Hospital and Chang Gung University, Taoyuan, Taiwan; ^5^Department of Dermatology, Xiamen Chang Gung Hospital, Xiamen, China; ^6^College of Medicine, Chang Gung University, Taoyuan, Taiwan

## Abstract

Adverse drug reactions (ADRs) remain a common and major problem in healthcare. Severe cutaneous adverse drug reactions (SCARs), such as Stevens–Johnson syndrome (SJS)/toxic epidermal necrolysis (TEN) with mortality rate ranges from 10% to more than 30%, can be life threatening. A number of recent studies demonstrated that ADRs possess strong genetic predisposition. ADRs induced by several drugs have been shown to have significant associations with specific alleles of human leukocyte antigen (HLA) genes. For example, hypersensitivity to abacavir, a drug used for treating of human immunodeficiency virus (HIV) infection, has been proposed to be associated with allele 57:01 of *HLA-B* gene (terms *HLA-B*^∗^*57:01*). The incidences of abacavir hypersensitivity are much higher in Caucasians compared to other populations due to various allele frequencies in different ethnic populations. The antithyroid drug- (ATDs- ) induced agranulocytosis are strongly associated with two alleles: *HLA-B*^∗^*38:02* and *HLA-DRB1*^∗^*08:03*. In addition, *HLA-B*^∗^*15:02* allele was reported to be related to carbamazepine-induced SJS/TEN, and *HLA-B*^∗^*57:01* in abacavir hypersensitivity and flucloxacillin induced drug-induced liver injury (DILI). In this review, we summarized the alleles of HLA genes which have been proposed to have association with ADRs caused by different drugs.

## 1. Introduction

Major histocompatibility complex (MHC) are a group of cell surface proteins that can bind to foreign molecules in order to be recognized by corresponding T cells followed by inducting immune systems. MHC is highly conserved and presents in all vertebrate species. In human, MHC is also known as human leukocyte antigen (HLA) complex, which consists more than 200 genes on chromosome 6 and can be categorized into three subgroups: class I, class II, and class III. Class I MHC, being recognized by CD8+ T cells, consists of three main genes, that is, *HLA-A*, *HLA-B*, and *HLA-C*. Class II MHC, being recognized by CD4+ T cells, consists of 6 main genes, that is, *HLA-DPA1*, *HLA-DPB1*, *HLA-DQA1*, *HLA-DQB1*, *HLA-DRA*, and *HLA-DRB1*.

HLA class I molecules are expressed in almost all the cells and are responsible for presenting peptides to immune cells. Generally, old proteins in the cells will be broken down consistently in order to synthesize new peptides. Some of these broken peptide pieces attach to the MHC molecules and are further recognized by immune cells as “self.” In another situation, if a cell is infected by pathogens, pathogenic peptides attached to MHC molecules will be recognized as “nonself” and further trigger the downstream immune responses against the antigens [1]. *HLA* genes are found to be numerous and highly polymorphic in order to bind various kinds of peptides originated from self or foreign antigens. A total of more than 1500 alleles of *HLA-B* gene have been identified [2]. Variations in the *HLA* genes play an important role in determining the susceptibility to autoimmune disease and infections; they are also critical in the field of transplant surgery where the donors and the recipients must be HLA-compatible [3].

In rare cases, some drugs are capable of inducing immune responses through interactions with MHC molecules, known as adverse drug reactions (ADRs). ADRs are one of the most common causes of hospitalization and mortality in healthcare. The definition of an ADR has been changed from time to time. In the 1970s, the World Health Organization (WHO) has first defined that an ADR is “a response to a drug that is noxious and unintended and occurs at doses normally used in man for the prophylaxis, diagnosis or therapy of disease, or for modification of physiological function” [4]. However, in most cases, the ADRs might not result in effects as severe as harms or injuries like the word “noxious” addressed by WHO. Therefore, Edwards and Aronson [5] suggested to use an alternative definition; that is, an ADR is “an appreciably harmful or unpleasant reaction, resulting from an intervention related to the use of a medicinal product, which predicts hazard from future administration and warrants prevention or specific treatment, or alteration of the dosage regimen, or withdrawal of the product.” It is also defined as “an undesirable effect, reasonably associated with the use of the drug that may occur as a part of the pharmacological action of a drug or may be unpredictable in its occurrence.”

## 2. ADRs Associated with Immunological Reactions

Several studies showed that ADRs are a major public health problem worldwide, which account for about 6.5% of all hospitalizations in the United States, Canada, and the United Kingdom, and it also resulted in a mortality rate approximate to 0.13% [6–8]. ADRs could be categorized into 6 different types [5]. Among them, type B, also known as non-dose-related or bizarre, is unpredictable and results in a high mortality rate oftentimes. This type of ADRs is usually associated with immunological reactions involving different HLA alleles and resulted in skin injury, hepatic failure, or dramatically reduced numbers of white blood cells.

Skin injury includes various kinds of spectrum such as mild rash maculopapular exanthema (MPE), fixed drug eruption (FDE), acute generalized exanthematous pustulosis (AGEP), and life-threatening severe cutaneous adverse drug reactions (SCARs) including drug reactions with eosinophilia and systemic symptoms (DRESS), Stevens–Johnson syndrome (SJS), and toxic epidermal necrolysis (TEN) [9]. Patients who developed MPE are usually observed with generalized, widespread mild skin rashes with red macular (not elevated) or papular (elevated) eruptions. FDE can be diagnosed by observing one or more local annular or oval erythematous patches without hyperpigmentation. The term “fixed” is considered as its recurrent lesion due to reexposure of the same culprit drug, and the lesions always occur at the same locations on the skin. AGEP is a rare, acute eruption characterized by the rapid development of many numerous pustules, which are nonfollicular sterile pustules, and is located in the epidermis. Fever, leukocytosis, and eosinophilia are usually present in AGEP patients.

SJS, SJS, and TEN overlap (SJS/TEN) and TEN are classified as the same disease spectrum with increasing severity and with extent of widespread epidermal detachment, known as SCARs [10]. All of them usually present with a variety of skin lesions, including patches, atypical targetoid macules, and erythematous or violaceous macules. In addition, SJS/TEN often has mucocutaneous involvement, which is the characteristic feature of SJS/TEN. In addition, the oral mucosa is more commonly involved than the ocular, genital, or anal mucosa. The degree of skin detachments of SJS, SJS/TEN, and TEN are defined as less than 10%, 10–30%, and 30% of body surface area, respectively. Full-thickness epidermal necrosis is a typical pathological feature of SJS/TEN. The clinical characteristics of DRESS are different from SJS/TEN. DRESS usually presents less or no skin detachment and no mucocutaneous involvement but with more internal organ involvement and hematological abnormalities such as typical eosinophilia, atypical lymphocytes, hepatitis, and high fever with frequent reactivation of human herpesvirus. Histopathological characters of DRESS are epidermal spongiosis, dyskeratosis, and interface vacuolization.

Other than skin injury, hepatic failure, such as drug-induced liver injury (DILI), is rare but life threatening. DILI is different from drug overdose toxicity in which the risk and severity of such kind of liver injury usually increases with the dose taken. DILI accounts for 7–15% of the cases of acute liver failure in Europe and the United States [11–13]. Up to 10% of DILI can progress to acute liver failure in the US and European studies, and the incidence is estimated to be 2.4 per 100,000 person-years (in a retrospective population-based study of 1.64 million UK subjects) [14] to 13.9 per 100,000 inhabitants (in a prospective analysis in France) [15].

Agranulocytosis, also known as agranulosis or granulopenia, is an acute condition involving a severe and dramatic decreasing of white blood cell counts, which is life threatening. It is recently reported to be induced by antithyroid drugs in rare situations and is associated with HLA alleles [16].

## 3. Hypothesis of Immune Response

Drugs or its reactive metabolites are considered as foreign antigens that bind to T cell receptors (TCR) and further activate immune response. Four hypotheses have been proposed to explain how the immune system is activated in a HLA molecule-dependent manner: (i) the “hapten/prohapten” theory, (ii) the “p-i” concept, (iii) the “altered peptide repertoire” model, and (iv) the “altered TCR repertoire” model [17, 18].

The “hapten/prohapten” theory proposes that a drug or its reactive metabolite may bind covalently to an endogenous peptide to form an antigenic hapten-carrier complex. In this model, the covalent bonds are established among the drug (or its metabolite), self-peptides, and HLA molecule. It then results in the induction of drug-specific immune responses.

The “pharmacological interaction with immune receptors (p-i)” concept postulates that a drug or its reactive metabolite may directly, reversibly, and noncovalently bind to the HLA and/or TCR without binding to the antigenic peptide. In this “p-i” model, the classic antigen-processing pathway in antigen-presenting cells may be bypassed.

The “altered peptide repertoire” model proposes that a drug could strongly bind to the self-peptide repertoire and alter the conformation of this peptide repertoire presented to HLA and TCR. In the “altered peptide repertoire” model, the drug may not directly bind to HLA.

Finally, the “altered TCR repertoire” model suggests that the drug (e.g., sulfamethoxazole) binds to the specific TCR and alters the conformation of TCR, which has the potential to bind a HLA-self peptide complex to elicit immune reaction. In the “altered TCR repertoire” model, the TCR serves as an initial drug interaction molecular. With the binding of an offending drug presented to the HLA molecule or TCR, the HLA-drug-TCR complex may trigger a series of activations of cell signaling and result in an expansion of cytotoxic T lymphocytes (CTL), cytotoxic protein secretions, and keratinocyte death in patients with SJS/TEN. A recent study has shown the importance of TCR in the pathogenic mechanism of SJS/TEN onset by clarifying the shared and restricted TCR use in carbamazepine-induced SJS/TEN patients [19]. Additionally, another interesting study demonstrated that the endogenous peptide-bound *HLA-B*^∗^*15:02* molecule presents carbamazepine to TCR of CTL to initiate the immune reactions in carbamazepine-induced SJS/TEN [20].

## 4. Drugs and HLA Alleles

A couple of drugs have been proposed to induce *HLA*-associated ADRs (Table 1). In this section, we summarized some of the well-known drugs and the *HLA* alleles associated with ADRs induced by these drugs. For more detailed information, please see the list in Table 1.

### 4.1. Abacavir Hypersensitivity and *HLA-B*^∗^*57:01* (Skin)

Abacavir is a nucleotide reverse transcriptase inhibitor used as part of adjuvant therapy in human immunodeficiency virus- (HIV-) infected patients. In 5–8% of treated patients, abacavir can cause hypersensitivity responses. More than 90% of the patients with hypersensitive syndrome start within 6 weeks of treatment and require immediate cessation of the medication. Re-exposure of the abacavir leads to rapid appearance of symptoms and higher chance to induce more severe symptoms [21]. Symptoms reported including fever, rash, malaise/fatigue, and gastrointestinal symptoms such as nausea, vomiting, and diarrhea. Respiratory symptoms occurred in 30% of cases including dyspnea, cough, and pharyngitis. In very rare cases, abacavir might result in more severe reaction such as SJS/TEN [22, 23].

In 2002, two publications first proposed that abacavir hypersensitivity was significantly associated with the presence of allele *HLA-B*^∗^*57:01* in Australian and British cohorts [24, 25]. Saag et al. [26] further demonstrated that there is a higher chance of developing hypersensitivity in Caucasians than African-Americans who were treated with abacavir. Among those suffered from abacavir hypersensitivity, 44% of Caucasians and 100% of African-Americans showed positive of *HLA-B*^∗^*57:01* allele. Recently, *HAL-B*^∗^*57:01* was screened in other populations (summarized in Martin et al. [27]). In general, the frequency of *HLA-B*^∗^*57:01* allele is much higher in Caucasians than in other populations. In Taiwan, abacavir hypersensitivity is less frequent, as it occurs in approximately 0.3% of HIV-infected patients who undergo abacavir-containing combination antiretroviral therapy (a total of 320 patients studied). The possible reason might be the low frequency of the *HLA-B*^∗^*5701* allele in Taiwanese population [28].

The mechanisms of abacavir hypersensitivity is better studied compared to other drug-induced ADRs. It is thought that short peptide fragments, derived from either the drug or its metabolites, form a peptide-HLA complex specifically with *HLA-B*^∗^*57:01*. This complex activates CD8+ T cells, which release inflammatory cytokines and start the hypersensitivity response. More recently, it has been shown that abacavir might occupy a space below the region of HLA that presents peptides, which leads to an altered peptide presentation and trigger an autoimmune reaction [29]. By using X-ray crystallography and structural analysis, Yerly et al. further proposed that the hypersensitivity reaction is due to both types of T cells that recognize self-peptide/*HLA-B*^∗^*57:01* complexes and cross react with viral peptide/*HLA-B*^∗^*57:01* complexes due to similarity in drug-specific T cell receptors contact residues [30].

As genetic screens for *HLA-B*^∗^*57:01* could significantly reduce the incidence of abacavir hypersensitivity in Caucasians, the European Medicines Agency and US Food and Drug Administration (FDA) recommend prospective screening for *HLA-B*^∗^*57:01* for patients who are considered to undergo abacavir treatment [27, 31].

### 4.2. Carbamazepine and Oxcarbazepine Hypersensitivity and *HLA-B*^∗^*15:02*, *HLA-B*^∗^*15:11*, and *HLA-A*^∗^*31:01* (Skin)

Carbamazepine is an important drug used in the treatment of epilepsy, trigeminal neuralgia, and bipolar disorder [32–34]. In 2004, Carbamazepine was first reported to be strongly associated with allele *HLA-B*^∗^*15:02* by studying patients developed SJS/TEN in Taiwan (OR > 1000) [35]. This association was validated in different populations, including those in Thailand, Malaysia, Singapore, and India [36–38]. A large scale of prospective study including almost 5000 participants from 23 hospitals in Taiwan showed that 7.7% of the subjects are *HLA-B*^∗^*15:02* positive. These subjects carrying *HLA-B*^∗^*15:02* were then advised to take alternative drugs other than carbamazepine [39]. Consequently, taking the alternative drug greatly reduced the change of developing SCARs, especially SJS/TEN. Based on the findings from these studies, the genetic screening of *HLA-B*^∗^*15:02* prior to the use of carbamazepine for certain Asian populations are recommended by different health regulatory agencies [40].

*HLA-B* ^∗^ *15:02* association and carbamazepine-induced SJS/TEN is also proposed to be ethnic specific. It is likely due to different genetic background as the allele frequency varies among different populations. It is relatively high in Han Chinese (0.057–0.145), Malaysians (0.12–0.157), and Thai (0.085–0.275) compared to Japanese (0.002), Koreans (0.004), and Europeans (0.01–0.02) [41–47].

In the populations with lower frequency of *HLA-B*^∗^*15:02*, that is, Northern Europeans, Japanese, and Koreans, more recent genome-wide association studies (GWAS) showed that *HLA-A*^∗^*31:01* allele has relatively stronger association with carbamazepine-induced hypersensitivity (OR = 25.93, 10.8, and 7.3 in the three populations, resp.) [41, 48–50]. In addition, *HLA-B*^∗^*15:11* allele was shown to be associated with carbamazepine-induced SJS/TEN in Japanese and Korean populations as well (OR = 9.8 and 18.1 in the two populations, resp.) [41, 47]. The different strength and specificity of HLA association with carbamazepine-induced SCARs further suggest that it is necessary to perform different genetic tests for different populations.

Oxcarbazepine is also an important drug used in the treatment of epilepsy. Oxcarbazepine-induced cutaneous ADRs presented with less clinical severity including limited skin detachment (all ≦ 5%) and no mortality compared to carbamazepine. Therefore, it is commonly used as an alternative to carbamazepine. A most recent study which enrolled 50 patients in Taiwan and Thailand from 2006 to 2014 identified a significant association between *HLA-B*^∗^*15:02* and SJS/TEN (OR = 27.90; *P* = 1.87 × 10^−10^). The results of study suggested that although oxcarbazepine is used as an alternative due to the less severity of drug reactions, genetic test should also be considered further, particularly for the populations with higher frequency of *HLA-B*^∗^*15:02* [51].

### 4.3. Allopurinol Hypersensitivity and *HLA-B*^∗^*58:01* (Skin)

Allopurinol is a xanthine oxidase inhibitor used in the treatment of gout and hyperuricemia. A study comparing the data in 2005 and in 2011 from Taiwan's National Health Insurance Research Database, which belongs to the nationwide population database with more than 23 million insured enrollees, demonstrated that allopurinol hypersensitivity happened in about 0.4% of the new users every year. About half of them required hospitalization [52]. Patients who underwent hospitalization had very high mortality rate (0.39/1000 new users). In 2005, the first case-control study in Taiwan showed that *HLA-B*^∗^*58:01* allele is the genetic marker of allopurinol-induced SCARs in Han Chinese (OR = 580.3, *P* = 4.7 × 10^−24^) [53]. This association was then validated in different populations, such as Thailand, Japan, South Korea, Hong Kong, Australia, Portugal, and Europe [54–59]. Currently, *HLA-B*^∗^*58:01* is considered as a useful genetic marker for allopurinol-SCARs in multiple ethnic populations worldwide [31]. The American College of Rheumatology guideline thus recommends the *HLA-B*^∗^*58:01* genetic screening for allopurinol new users in Asia populations since 2012.

A most recent study in Taiwan further enrolled a large number of patients with allopurinol-induced ADRs in order to investigate the associations between *HLA-B*^∗^*58:01*, renal function, gene dosage, and drug dosage with the risk of allopurinol-induced ADRs development [58]. The authors showed that *HLA-B*^∗^*58:01* was strongly associated with ADRs (OR = 44.0; *P* = 2.6 × 10^−41^) and was also highly correlated with disease severity. That is, patients carrying *HLA-B*^∗^*58:01* had much higher chance to develop SCARs comparing to MPE, particularly those individuals with homozygous *HLA-B*^∗^*58:01*. Furthermore, coexistence of *HLA-B*^∗^*58:01* and renal impairment increased the risk and predictive accuracy of allopurinol-induced ADRs. This study suggests that patients with the coexistence of *HLA-B*^∗^*58:01* and renal impairment should be cautious and avoid to use allopurinol.

### 4.4. Dapsone Hypersensitivity and *HLA-B*^∗^*13:01* (Skin)

Dapsone alone or in-combination with other drugs are effective for the treatment or prevention of infectious diseases (e.g. leprosy, malaria and pneumocystis pneumonia). However, about 0.5–3.6% of persons who were treated with dapsone developed hypersensitivity reactions. A recent genome-wide association study involving 872 participants (39 participants showed dapsone hypersensitivity syndrome and 833 controls) identified that SNP rs2844573, located between the HLA-B and MICA loci, was significantly associated with the dapsone hypersensitivity (OR = 6.18; *P* = 3.84 × 10^−13^) [60]. The authors further confirmed that *HLA-B*^∗^*13:01* is associated with the dapsone hypersensitivity (OR = 20.53; *P* = 6.84 × 10^−25^). The allele showed a sensitivity of 85.5% and a specificity of 85.7% for dapsone hypersensitivity from this study and thus can be used as a marker of dapsone hypersensitivity. However, the hypersensitivity has not been studied in other ethnic populations.

### 4.5. Amoxicillin-Clavulanate-Induced DILI and *HLA* Haplotypes

Amoxicillin-clavulanate (AC) is one of the most commonly prescribed antimicrobial drugs worldwide. However, it is a known cause of DILI and accounts for 10–13% of hospitalizations. Hautekeete et al. first reported a strong association between HLA and AC-induced DILI in Europeans [61]. The authors observed a much higher frequency of *DRB1*^∗^*15:01*-*DRB5*^∗^*01:01*-*DQB1*^∗^*06:02* haplotype in patients with AC-induced DILI compared to normal healthy controls (57.1% in cases versus 11.7% in controls, *P* < 10^−6^). The association was further validated in two UK populations (OR = 2.3 and 9.3 for the two populations, resp.) [62, 63]. A recent study by performing GWAS in 201 patients further confirmed the association of AC-induced DILI with *DRB1*^∗^*15:01* allele (OR = 4.2; *P* = 4.6 × 10^−10^) [64]. In addition, the study further identified two novel HLA alleles as risk factors of AC-induced DILI: *HLA*-*A*^∗^*02:01* in all patients (OR = 2.2; *P* = 1.8 × 10^−10^) and *HLA-B*^∗^*18:01* with nominal significance independently of *HLA*-*A*^∗^*02:01* and *HLA*-*DQB1*^∗^*06:02* in Spanish patients only.

### 4.6. Flucloxacillin-Induced DILI and *HLA-B*^∗^*57:01* Association

Flucloxacillin is an antibiotic belonging to penicillin class and is used widely for the treatment for staphylococcal infection in Europe. Flucloxacillin is a common cause of DILI and is also reported to be associated with cholestatic liver disease. A GWAS study enrolling 51 patients showed a strong association between flucloxacillin-induced DILI and a marker, rs2395029[G]. This marker is in complete linkage disequilibrium with *HLA-B*^∗^*5701* (*P* = 8.7 × 10^−33^) [65]. The authors further performed MHC genotyping and confirmed the association of flucloxacillin induced DILI with *HLA-B*^∗^*5701* (OR = 80.6, *P* = 9.0 × 10^−19^). This is an interesting finding because *HLA-B*^∗^*57:01* is also associated with abacavir hypersensitivity, but these patients were not reported to develop liver injury. It still remains unclear whether it resulted from the binding of different drugs/metabolites to the same HLA allele and subsequent initiation of immune responses or it is merely a coincidental event. As the positive predictive value of *HLA-B*^∗^*57:01* is as low as 0.12% [31], the genetic screening for *HLA-B*^∗^*57:01* before the prescription of flucloxacillin to new users may not be clinically relevant.

### 4.7. Antithyroid Drug-Induced Agranulocytosis and *HLA-B*^∗^*38:02*-*HLA-DRB1*^∗^*08:03* Haplotype

Other than skin and liver injures, drug reactions could also affect the immune system directly. Antithyroid drugs (ATDs) have been the cornerstones treatment of Graves' disease (GD), which is the leading cause of hyperthyroidism. It has been reported that ATDs may induce agranulocytosis resulting in lower number of white blood cells and is likely to be life threatening. However, the genetic risk factors have not been identified until recently. Chen et al. conducted both classic genotyping and GWAS to elucidate the genetic association between ATD-induced agranulocytosis and HLA genes in Taiwan [16]. First of all, they performed direct HLA genotyping including 6 classical loci for a total of 42 agranulocytosis cases and about 1200 GD controls. The results showed strong associations of ATD-induced agranulocytosis with two alleles: *HLA-B*^∗^*38:02* (*P* = 6.75 × 10^−32^) and *HLA-DRB1*^∗^*08:03* (*P* = 1.83 × 10^−9^), which are in independent LD blocks. From GWAS, two more markers were further identified in the genomic region of HLA genes (6q21): rs17193122 (*P* = 4.29 × 10^−27^), which is in LD block with *HLA-B*^∗^*38:02*, and rs116869525 (*P* = 1.27 × 10^−8^). The two markers are in the same LD block with *HLA-DRB1*^∗^*08:03*. The authors further showed that the patients who carried both alleles have much higher chance to develop agranulocytosis compared to those who had only one allele. This is an interesting finding similar to the observation in amoxicillin-clavulanate-induced DILI: class I and class II HLA confer genetic susceptibility to the same drug adverse effect, as we mentioned above.

## 5. Different Techniques Used to Screen HLA Alleles or Predict Hypersensitivity Reactions in New Drug Users

As the association between HLA alleles and the chance of developing SCARs has been shown in many studies, it is important and advised to have the genetic test for new users of the drugs mentioned above. Systematic and large-scale genetic testing is mostly available for *HLA-B*^∗^*57:01* through commercial laboratories in the US as this allele has the highest frequency in Caucasians. These kits typically offer single allele testing with a short turnaround time. The genotype results are either “positive” (*HLA-B*^∗^*57:01* being present in one or both copies of the *HLA-B* gene) or “negative” (no copies of *HLA-B*^∗^*57:01* are present). There are no intermediate phenotypes because *HLA-B* is expressed in a codominant manner [27]. Although most of the technologies were developed based on the purpose of detecting *HLA-B*^∗^*57:01* allele, the concept can also be applied to test other alleles. Therefore, in the following paragraph, we summarize the technologies developed to genotype HLA on the purpose of screening new drug users to avoid potential ADRs (Table 2).

### 5.1. PCR-Based Assays

Sequence-specific oligonucleotide (SSO) assays for HLA typing was one of the first PCR-based HLA typing methods [66, 67]. The technique amplifies a particular HLA gene locus such as HLA-A, HLA-B, or HLA-DRB1. Primers are generally designed in exons 2 and 3 for HLA class I and exon 2 for HLA class II—regions known to carry the most variations. Amplifications of all the alleles of a particular HLA locus can be performed in one PCR tube. PCR products of a particular HLA locus is then hybridized with labelled oligonucleutides specifically to a particular HLA allele or a group of alleles. Recently, several commercial kits have been developed based on SSO but can get the results in a shorter period of time such as LIFECODES HLA-B SSO Typing Kit (Immucor Transplant Diagnostics).

However, with the very high number of possible heterozygous HLA allele combinations, SSO is not sufficient to resolve all ambiguities as the method does not distinguish between *cis* and *trans* polymorphisms. Therefore, when the subjects are *HLA-B*^∗^*57* positive, sequence specific primer- (SSP-) PCR will be advised to further determine the specific genotype [68, 69]. Comparing to SSO, SSP-PCR has higher resolution and sensitivity as it uses sequence-specific primers.

More recently, new assays were developed for *HLA-B*^∗^*57*:*01* typing on a quantitative polymerase chain reaction (qPCR) platform [70–72]. This enables detection of primer specificity through differentiating Cq values by SYBR Green quantitative (q)PCR or analysis of allele-specific PCR by high-resolution melting. Implementation of these assays on a qPCR platform significantly decreases the processing and reaction time as well as reagent costs. Jung et al. further designed primers and probes based on DNA polymorphisms using hydrolysis probes (oftentimes referred to TaqMan technology) [73]. In their study, not only the primers but also the probes were designed for generating PCR products specifically from the *HLA-B*^∗^*57:01* allele. Although these primers may also generate products from other *HLA-B* alleles that do not induce hypersensitivity reactions, hydrolysis probes can differentiate these products and only give fluorescence signals if the *HLA-B*^∗^*57:01* allele is present. To reduce false-positive detection, additional probes are incorporated into a single multiplex reaction. The authors also developed PCR-restriction fragment length polymorphism (RFLP) assay for genotyping *HLA-B*^∗^*57:01*. In this assay, two pairs of primers, one specific to 57:01 allele and another pair is for control, selectively amplify genomic DNA followed by digestion with restriction enzymes NlaIII or RsaI. PCR products amplified from two different pairs of primers resulted in different sizes of fragments that can be visualized easily on the agarose gel.

### 5.2. Non-PCR-Based Techniques

Using monoclonal antibody to differentiate alleles *HLA-B*^∗^*57* and *HLA-B*^∗^*58* was first proposed by Kostenko et al. [74]. Monoclonal *HLA-B17* antibodies (mAb 3E12), which recognized both *HLA-B*^∗^*57* and *HLA-B*^∗^*58* allotypes (members of the group specificity, HLA-B17), was labelled with phycoerythrin while anti-CD45 labelled with FITC were incubated with blood from subjects. Lymphocytes were gated based upon scatter and CD45 bright expression, and mean fluorescence intensity for *HLA-B17* expression was then measured by flow cytometry. Although this is an inexpensive and rapid approach to detect the presence of two allotypes, it is proposed to be less specific and sensitive than PCR-based approaches. The subjects who test positive by mAb screening are recommended to proceed with high-resolution gold-standard typing, such as SSO and SSP-PCR, to ascertain the presence of *HLA-B*^∗^*5701* or *HLA-B*^∗^*5801*.

Patch testing is also used to predict hypersensitivity reaction of abacavir [75] and carbamazepine [76]. Giorgini and others performed patch testing on 100 subjects including 20 cases who had experienced a hypersensitivity reaction when treated with highly active antiretroviral therapy including abacavir. Among the cases with positive patch testing results, about 50% of them carry *HLA-B*^∗^*57:01* allele. More recently, Lin et al. proposed to use patch testing to predict carbamazepine induced hypersensitivity. They showed that about 60–70% of the cases who developed SJS/TEN and DRESS to carbamazepine had positive reactions in the patch testing. Although drug patch testing is a safe and inexpensive method for the identification of hypersensitivity, the sensitivity and specificity is not as good as PCR-based approaches.

## 6. Conclusion

In this review, we summarize the HLA alleles associated with ADRs induced by different drugs. From the literature, we learned that most of the HLA-associated ADRs have ethnic specificity. It is likely due to the different allele frequency between populations. Gonzalez-Galarza et al. summarize the allele frequencies of all the HLA genes and showed that the frequencies differ a lot [77]. For example, HLA-B^∗^57:01 has the highest frequency in Ireland, but has the lowest frequency in Cuba (African populations). The frequencies can of each allele in different populations be found in the public database (http://www.allelefrequencies.net/default.asp) [78].

Other than the HLA-associated ADRs being ethnic specific, there are also two interesting questions: (1) why one locus contributes to different ADRs as we have observed on *HLA-B*^∗^*57:01* in abacavir hypersensitivity and flucloxacillin-induced DILI. (2) How different loci contribute to the same ADRs as we have seen in antithyroid drug-induced agranulocytosis and amoxicillin-clavulanate-induced DILI. As class I and class II HLA genes have different structures, cell-type distributions, and functional roles in the immune system, the genetic susceptibility from both classes for a phenotype are expected to be intriguing. How both class I and class II HLA genes confer genetic susceptibility to the same ADR requires further pathophysiological investigations.

## Figures and Tables

**Table 1:** Drug-induced ADRs and HLA allele associations.

Drug	HLA allele	Phenotype	Population	Reference
Abacavir	B^∗^57:01	HSS	Australian	[25]
			African American	[26]
			Brazilian	[79]
			British	[24]
			Indian	[80]
			Iranian	[81]

Carbamazepine	B^∗^15:02	SJS/TEN	Taiwanese	[35, 82]
			Han Chinese	[83]
			Thai	[37]
			Malaysian	[44]
			Asian	[46]

	A^∗^31:01	MPE, HSS, SJS/TEN	Han Chinese	[49, 82]
			Caucasian	[48]
			Japanese	[50]

	B^∗^15:11	SJS/TEN	Japanese	[47, 84]

Allopurinol	B^∗^58:01	SJS/TEN	Han Chinese	[53]
			Caucasian	[57]
			Thai	[59]
			Japanese	[59]

		SCARS	Taiwanese	[85]

Dapsone	B^∗^13:01	HSS		[60]

Phenytoin	B^∗^15:02	SJS/TEN	Han Chinese	[83]
			Thai	[37]

Lamotrigine	A^∗^31:01	HSS	British	[86]
	B^∗^15:02	SJS/TEN	Han Chinese	[83]

Nevirapine	DRB1^∗^01:01	DRESS	Hispanics, African	[87]

	B^∗^14:02	HSS	Sardinian	[88, 89]

Sulphamethoxazole	B^∗^38	SJS/TEN	European	[57]

Methazolamide	B^∗^59:01, CW^∗^01:02	SJS/TEN	Korean and Japanese	[84]

Amoxicillin-clavulanate	DRB1^∗^15:01-DQB1^∗^06:02	DILI	Caucasian	[64]

Flucloxacillin	B^∗^57:01	DILI	Caucasian	[65]

Lumiracoxib	DRB1^∗^15:01	DILI	Not available	[90]

Ticlopidine	A^∗^33:03	DILI	Japanese	[91]

Antithyroid drugs	B^∗^38:02-DRB1^∗^08:03	Agranulocytosis	Taiwanese	[16]

**Table 2:** Available genetic tests.

Platform	Technology	Specificity	Advantage	References
PCR	Sequence specific oligonucleotides (SSO)	>95%	Commercial kits available	[66, 92]
PCR	sequence-specific primer (SSP) PCR	>97%	More specific than SSO	[66, 68, 69]
Real-time PCR	Hydrolysis probe (TaqMan)	>99%	Mismatch in the probe region seems to be more sensitive than those in the primer region	[73]
Flow cytometry	HLA-B17 specific monoclonal antibody	~80%		[93]
Patch testing		60–70%	Safe and inexpensive	[75, 76]

## Abbreviations

ADR: Adverse drug reactions
HLA: Human leukocyte antigen
DRESS: Drug reaction with eosinophilia and systemic symptoms
SCAR: Severe cutaneous adverse drug reactions
SJS: Stevens–Johnson syndrome
TEN: Toxic epidermal necrolysis.

## References

[1] Cresswell P., Ackerman A. L., Giodini A., Peaper D. R., Wearsch P. A. (2005). Mechanisms of MHC class I-restricted antigen processing and cross-presentation. *Immunological Reviews*.

[2] Shiina T., Hosomichi K., Inoko H., Kulski J. K. (2009). The HLA genomic loci map: expression, interaction, diversity and disease. *Journal of Human Genetics*.

[3] Bray R. A., Hurley C. K., Kamani N. R. (2008). National marrow donor program HLA matching guidelines for unrelated adult donor hematopoietic cell transplants. *Biology of Blood and Marrow Transplantation*.

[4] International drug monitoring (1972). The role of national centres. Report of a WHO meeting. *World Health Organization Technical Report Series*.

[5] Edwards I. R., Aronson J. K. (2000). Adverse drug reactions: definitions, diagnosis, and management. *The Lancet*.

[6] Lazarou J., Pomeranz B. H., Corey P. N. (1998). Incidence of adverse drug reactions in hospitalized patients: a meta-analysis of prospective studies. *JAMA*.

[7] Kvasz M., Allen I. E., Gordon M. J. (2000). Adverse drug reactions in hospitalized patients: a critique of a meta-analysis. *Medscape General Medicine*.

[8] Miguel A., Azevedo L. F., Araujo M., Pereira A. C. (2012). Frequency of adverse drug reactions in hospitalized patients: a systematic review and meta-analysis. *Pharmacoepidemiology and Drug Safety*.

[9] Falasca F., Dello Russo C., Mora B. (2016). Comparative analysis of real-time polymerase chain reaction methods to typing *HLA-b^∗^57:01* in HIV-1-positive patients. *AIDS Research and Human Retroviruses*.

[10] Chung W. H., Wang C. W., Dao R. L. (2016). Severe cutaneous adverse drug reactions. *The Journal of Dermatology*.

[11] Ostapowicz G., Fontana R. J., Schiodt F. V. (2002). Results of a prospective study of acute liver failure at 17 tertiary care centers in the united states. *Annals of Internal Medicine*.

[12] Russo M. W., Galanko J. A., Shrestha R., Fried M. W., Watkins P. (2004). Liver transplantation for acute liver failure from drug induced liver injury in the united states. *Liver Transplantation*.

[13] Bjornsson E., Jerlstad P., Bergqvist A., Olsson R. (2005). Fulminant drug-induced hepatic failure leading to death or liver transplantation in Sweden. *Scandinavian Journal of Gastroenterology*.

[14] de Abajo F. J., Montero D., Madurga M., Garcia Rodriguez L. A. (2004). Acute and clinically relevant drug-induced liver injury: a population based case-control study. *British Journal of Clinical Pharmacology*.

[15] Sgro C., Clinard F., Ouazir K. (2002). Incidence of drug-induced hepatic injuries: a French population-based study. *Hepatology*.

[16] Chen P. L., Shih S. R., Wang P. W. (2015). Genetic determinants of antithyroid drug-induced agranulocytosis by human leukocyte antigen genotyping and genome-wide association study. *Nature Communications*.

[17] Pichler W. J. (2002). Pharmacological interaction of drugs with antigen-specific immune receptors: the p-i concept. *Current Opinion in Allergy and Clinical Immunology*.

[18] Watkins S., Pichler W. J. (2013). Sulfamethoxazole induces a switch mechanism in T cell receptors containing TCRVΒ20-1, altering PHLA recognition. *PLoS One*.

[19] Ko T. M., Chung W. H., Wei C. Y. (2011). Shared and restricted T-cell receptor use is crucial for carbamazepine-induced Stevens-Johnson syndrome. *The Journal of Allergy and Clinical Immunology*.

[20] Wei C. Y., Chung W. H., Huang H. W., Chen Y. T., Hung S. I. (2012). Direct interaction between HLA-B and carbamazepine activates T cells in patients with Stevens-Johnson syndrome. *The Journal of Allergy and Clinical Immunology*.

[21] Hetherington S., McGuirk S., Powell G. (2001). Hypersensitivity reactions during therapy with the nucleoside reverse transcriptase inhibitor abacavir. *Clinical Therapeutics*.

[22] Bossi P., Roujeau J. C., Bricaire F., Caumes E. (2002). Stevens-Johnson syndrome associated with abacavir therapy. *Clinical Infectious Diseases*.

[23] Pahk R., Azu M. C., Taira B. R., Sandoval S. (2009). Antiretroviral-induced toxic epidermal necrolysis in a patient positive for human immunodeficiency virus. *Clinical and Experimental Dermatology*.

[24] Hetherington S., Hughes A. R., Mosteller M. (2002). Genetic variations in *HLA-B* region and hypersensitivity reactions to abacavir. *The Lancet*.

[25] Mallal S., Nolan D., Witt C. (2002). Association between presence of *HLA-B*^∗^*5701*, *HLA-DR7*, and *HLA-DQ3* and hypersensitivity to HIV-1 reverse-transcriptase inhibitor abacavir. *The Lancet*.

[26] Saag M., Balu R., Phillips E., Hughes A., Sutherland‐Phillips D., Mallal S., Shaefer M., Study of Hypersensitivity to Abacavir and Pharmacogenetic Evaluation Study Team (2008). High sensitivity of human leukocyte antigen-B^∗^5701 as a marker for immunologically confirmed abacavir hypersensitivity in white and black patients. *Clinical Infectious Diseases*.

[27] Martin M. A., Klein T. E., Dong B. J. (2012). Clinical pharmacogenetics implementation consortium guidelines for *HLA-B* genotype and abacavir dosing. *Clinical Pharmacology & Therapeutics*.

[28] Sun H. Y., Hung C. C., Lin P. H. (2007). Incidence of abacavir hypersensitivity and its relationship with HLA-B^∗^5701 in HIV-infected patients in Taiwan. *Journal of Antimicrobial Chemotherapy*.

[29] Pirmohamed M., Ostrov D. A., Park B. K. (2015). New genetic findings lead the way to a better understanding of fundamental mechanisms of drug hypersensitivity. *The Journal of Allergy and Clinical Immunology*.

[30] Yerly D., Pompeu Y., Schutte R. (2017). Structural elements recognized by abacavir-induced T cells. *International Journal of Molecular Sciences*.

[31] Yip V. L. M., Alfirevic A., Pirmohamed M. (2015). Genetics of immune-mediated adverse drug reactions: a comprehensive and clinical review. *Clinical Reviews in Allergy & Immunology*.

[32] Marson A. G., Al-Kharusi A. M., Alwaidh M. (2007). The SANAD study of effectiveness of carbamazepine, gabapentin, lamotrigine, oxcarbazepine, or topiramate for treatment of partial epilepsy: an unblinded randomised controlled trial. *The Lancet*.

[33] Post R. M., Ketter T. A., Uhde T., Ballenger J. C. (2007). Thirty years of clinical experience with carbamazepine in the treatment of bipolar illness: principles and practice. *CNS Drugs*.

[34] Cruccu G., Gronseth G., Alksne J. (2008). AAN-EFNS guidelines on trigeminal neuralgia management. *European Journal of Neurology*.

[35] Chung W. H., Hung S. I., Hong H. S. (2004). Medical genetics: a marker for Stevens-Johnson syndrome. *Nature*.

[36] Tangamornsuksan W., Chaiyakunapruk N., Somkrua R., Lohitnavy M., Tassaneeyakul W. (2013). Relationship between the *HLA-B*^∗^*1502* allele and carbamazepine-induced Stevens-Johnson syndrome and toxic epidermal necrolysis: a systematic review and meta-analysis. *JAMA Dermatology*.

[37] Locharernkul C., Loplumlert J., Limotai C. (2008). Carbamazepine and phenytoin induced Stevens-Johnson syndrome is associated with HLA-B^∗^1502 allele in Thai population. *Epilepsia*.

[38] Mehta T. Y., Prajapati L. M., Mittal B. (2009). Association of HLA-B^∗^1502 allele and carbamazepine-induced *Stevens-Johnson syndrome* among Indians. *Indian Journal of Dermatology, Venereology and Leprology*.

[39] Chen P., Lin J. J., Lu C. S. (2011). Carbamazepine-induced toxic effects and HLA-B^∗^1502 screening in Taiwan. *The New England Journal of Medicine*.

[40] Ferrell P. B., McLeod H. L. (2008). Carbamazepine, *HLA-B*^∗^*1502* and risk of Stevens-Johnson syndrome and toxic epidermal necrolysis: US FDA recommendations. *Pharmacogenomics*.

[41] Kim S. H., Lee K. W., Song W. J., Min K. U., Chang Y. S., Adverse Drug Reaction Research Group in Korea (2011). Carbamazepine-induced severe cutaneous adverse reactions and HLA genotypes in Koreans. *Epilepsy Research*.

[42] Kano Y., Hirahara K., Asano Y., Shiohara T. (2008). HLA-B allele associations with certain drugs are not confirmed in Japanese patients with severe cutaneous drug reactions. *Acta Dermato-Venereologica*.

[43] Aggarwal R., Sharma M., Modi M., Garg V. K., Salaria M. (2014). HLA-B^∗^ 1502 is associated with carbamazepine induced Stevens-Johnson syndrome in North Indian population. *Human Immunology*.

[44] Chang C. C., Too C. L., Murad S., Hussein S. H. (2011). Association of HLA-B^∗^1502 allele with carbamazepine-induced toxic epidermal necrolysis and Stevens-Johnson syndrome in the multi-ethnic Malaysian population. *International Journal of Dermatology*.

[45] He X. J., Jian L. Y., He X. L. (2013). Association between the *HLA-B*^∗^15:02 allele and carbamazepine-induced Stevens-Johnson syndrome/toxic epidermal necrolysis in HAN individuals of Northeastern China. *Pharmacological Reports*.

[46] Alfirevic A., Jorgensen A. L., Williamson P. R., Chadwick D. W., Park B. K., Pirmohamed M. (2006). *HLA-B* locus in Caucasian patients with carbamazepine hypersensitivity. *Pharmacogenomics*.

[47] Kaniwa N., Saito Y., Aihara M. (2010). *HLA-B*
^∗^
*1511* is a risk factor for carbamazepine-induced Stevens-Johnson syndrome and toxic epidermal necrolysis in Japanese patients. *Epilepsia*.

[48] McCormack M., Alfirevic A., Bourgeois S. (2011). HLA-A^∗^3101 and carbamazepine-induced hypersensitivity reactions in Europeans. *The New England Journal of Medicine*.

[49] Genin E., Chen D. P., Hung S. I. (2014). HLA-A^∗^31:01 and different types of carbamazepine-induced severe cutaneous adverse reactions: an international study and meta-analysis. *The Pharmacogenomics Journal*.

[50] Ozeki T., Mushiroda T., Yowang A. (2011). Genome-wide association study identifies *HLA-A*^∗^*3101* allele as a genetic risk factor for carbamazepine-induced cutaneous adverse drug reactions in Japanese population. *Human Molecular Genetics*.

[51] Chen C. B., Hsiao Y. H., Wu T. (2017). Risk and association of HLA with oxcarbazepine-induced cutaneous adverse reactions in Asians. *Neurology*.

[52] Yang C. Y., Chen C. H., Deng S. T. (2015). Allopurinol use and risk of fatal hypersensitivity reactions: a nationwide population-based study in Taiwan. *JAMA Internal Medicine*.

[53] Hung S. I., Chung W. H., Liou L. B. (2005). HLA-B^∗^5801 allele as a genetic marker for severe cutaneous adverse reactions caused by allopurinol. *Proceedings of the National Academy of Sciences of the United States of America*.

[54] Kang H. R., Jee Y. K., Kim Y. S. (2011). Positive and negative associations of HLA class I alleles with allopurinol-induced scars in Koreans. *Pharmacogenetics and Genomics*.

[55] Kaniwa N., Saito Y., Aihara M. (2008). HLA-B locus in Japanese patients with anti-epileptics and allopurinol-related Stevens–Johnson syndrome and toxic epidermal necrolysis. *Pharmacogenomics*.

[56] Lee M. H., Stocker S. L., Anderson J. (2012). Initiating allopurinol therapy: do we need to know the patient’s human leucocyte antigen status?. *Internal Medicine Journal*.

[57] Lonjou C., Borot N., Sekula P. (2008). A European study of HLA-B in Stevens-Johnson syndrome and toxic epidermal necrolysis related to five high-risk drugs. *Pharmacogenetics and Genomics*.

[58] Ng C. Y., Yeh Y. T., Wang C. W. (2016). Impact of the *HLA-B*^∗^*58:01* allele and renal impairment on allopurinol-induced cutaneous adverse reactions. *The Journal of Investigative Dermatology*.

[59] Tassaneeyakul W., Jantararoungtong T., Chen P. (2009). Strong association between *HLA-B*^∗^*5801* and allopurinol-induced Stevens-Johnson syndrome and toxic epidermal necrolysis in a Thai population. *Pharmacogenetics and Genomics*.

[60] Zhang F. R., Liu H., Irwanto A. (2013). *HLA-B*
^∗^
*13:01* and the dapsone hypersensitivity syndrome. *The New England Journal of Medicine*.

[61] Hautekeete M. L., Horsmans Y., Van Waeyenberge C. (1999). HLA association of amoxicillin-clavulanate–induced hepatitis. *Gastroenterology*.

[62] Donaldson P. T., Daly A. K., Henderson J. (2010). Human leucocyte antigen class II genotype in susceptibility and resistance to co-amoxiclav-induced liver injury. *Journal of Hepatology*.

[63] O'Donohue J., Oien K. A., Donaldson P. (2000). Co-amoxiclav jaundice: clinical and histological features and HLA class II association. *Gut*.

[64] Lucena M. I., Molokhia M., Shen Y. (2011). Susceptibility to amoxicillin-clavulanate-induced liver injury is influenced by multiple HLA class I and II alleles. *Gastroenterology*.

[65] Daly A. K., Donaldson P. T., Bhatnagar P. (2009). *HLA-B*
^∗^
*5701* genotype is a major determinant of drug-induced liver injury due to flucloxacillin. *Nature Genetics*.

[66] Dunckley H. (2012). HLA typing by SSO and SSP methods. *Methods in Molecular Biology*.

[67] Wordsworth B. P., Allsopp C. E., Young R. P., Bell J. I. (1990). HLA-DR typing using DNA amplification by the polymerase chain reaction and sequential hybridization to sequence-specific oligonucleotide probes. *Immunogenetics*.

[68] Martin A. M., Nolan D., Mallal S. (2005). HLA-B^∗^5701 typing by sequence-specific amplification: validation and comparison with sequence-based typing. *Tissue Antigens*.

[69] Olerup O., Zetterquist H. (1992). HLA-DR typing by PCR amplification with sequence-specific primers (PCR-SSP) in 2 hours: an alternative to serological DR typing in clinical practice including donor-recipient matching in cadaveric transplantation. *Tissue Antigens*.

[70] Stocchi L., Cascella R., Zampatti S., Pirazzoli A., Novelli G., Giardina E. (2012). The pharmacogenomic HLA biomarker associated to adverse abacavir reactions: comparative analysis of different genotyping methods. *Current Genomics*.

[71] Dello Russo C., Lisi L., Lofaro A. (2011). Novel sensitive, specific and rapid pharmacogenomic test for the prediction of abacavir hypersensitivity reaction: *HLA-B*^∗^*57:01* detection by real-time PCR. *Pharmacogenomics*.

[72] Hammond E., Mamotte C., Nolan D., Mallal S. (2007). *HLA-B*
^∗^
*5701* typing: evaluation of an allele-specific polymerase chain reaction melting assay. *Tissue Antigens*.

[73] Jung H. S., Tsongalis G. J., Lefferts J. A. (2017). Development of *HLA-B*^∗^*57:01* genotyping real-time PCR with optimized hydrolysis probe design. *The Journal of Molecular Diagnostics*.

[74] Kostenko L., Kjer-Nielsen L., Nicholson I. (2011). Rapid screening for the detection of HLA-B57 and HLA-B58 in prevention of drug hypersensitivity. *Tissue Antigens*.

[75] Giorgini S., Martinelli C., Tognetti L. (2011). Use of patch testing for the diagnosis of abacavir-related hypersensitivity reaction in HIV patients. *Dermatologic Therapy*.

[76] Lin Y.-T., Chang Y.-C., Hui R. C.-Y. (2013). A patch testing and cross-sensitivity study of carbamazepine-induced severe cutaneous adverse drug reactions. *Journal of the European Academy of Dermatology and Venereology*.

[77] Gonzalez-Galarza F. F., Takeshita L. Y. C., Santos E. J. M. (2015). Allele frequency net 2015 update: new features for HLA epitopes, KIR and disease and HLA adverse drug reaction associations. *Nucleic Acids Research*.

[78] Ghattaoraya G. S., Dundar Y., González-Galarza F. F. (2016). A web resource for mining HLA associations with adverse drug reactions: HLA-ADR. *Database*.

[79] Crovella S., Biller L., Santos S. (2011). Frequency of HLA B^∗^5701 allele carriers in abacavir treated-HIV infected patients and controls from northeastern Brazil. *Clinics*.

[80] Chakravarty J., Sharma S., Johri A., Chourasia A., Sundar S. (2016). Clinical abacavir hypersensitivity reaction among children in India. *Indian Journal of Pediatrics*.

[81] Baniasadi S., Shokouhi S. B., Tabarsi P. (2016). Prevalence of HLA-B^∗^5701 and its relationship with abacavir hypersensitivity reaction in Iranian HIV-infected patients. *Tanaffos*.

[82] Hung S. I., Chung W. H., Jee S. H. (2006). Genetic susceptibility to carbamazepine-induced cutaneous adverse drug reactions. *Pharmacogenetics and Genomics*.

[83] Man C. B., Kwan P., Baum L. (2007). Association between HLA-B^∗^1502 allele and antiepileptic drug-induced cutaneous reactions in Han Chinese. *Epilepsia*.

[84] Kim S. H., Kim M., Lee K. W. (2010). *HLA-B*
^∗^
*5901* is strongly associated with methazolamide-induced Stevens–Johnson syndrome/toxic epidermal necrolysis. *Pharmacogenomics*.

[85] Ko T. M., Tsai C. Y., Chen S. Y. (2015). Use of HLA-B^∗^58:01 genotyping to prevent allopurinol induced severe cutaneous adverse reactions in Taiwan: national prospective cohort study. *BMJ*.

[86] McCormack M., Urban T. J., Shianna K. V. (2012). Genome-wide mapping for clinically relevant predictors of lamotrigine- and phenytoin-induced hypersensitivity reactions. *Pharmacogenomics*.

[87] Martin A. M., Nolan D., James I. (2005). Predisposition to nevirapine hypersensitivity associated with HLA-DRB1^∗^0101 and abrogated by low CD4 T-cell counts. *AIDS*.

[88] Littera R., Carcassi C., Masala A. (2006). HLA-dependent hypersensitivity to nevirapine in Sardinian HIV patients. *AIDS*.

[89] Arnaldo P., Thompson R. E., Lopes M. Q., Suffys P. N., Santos A. R. (2013). Frequencies of cytochrome P450 2B6 and 2C8 allelic variants in the Mozambican population. *The Malaysian Journal of Medical Sciences*.

[90] Singer J. B., Lewitzky S., Leroy E. (2010). A genome-wide study identifies HLA alleles associated with lumiracoxib-related liver injury. *Nature Genetics*.

[91] Hirata K., Takagi H., Yamamoto M. (2007). Ticlopidine-induced hepatotoxicity is associated with specific human leukocyte antigen genomic subtypes in Japanese patients: a preliminary case-control study. *The Pharmacogenomics Journal*.

[92] Ng J., Hurley C. K., Baxter-Lowe L. A. (1993). Large-scale oligonucleotide typing for HLA-DRB1/3/4 and HLA-DQB1 is highly accurate, specific, and reliable. *Tissue Antigens*.

[93] De Spiegelaere W., Philippe J., Vervisch K. (2015). Comparison of methods for in-house screening of *HLA-B*^∗^*57:01* to prevent abacavir hypersensitivity in HIV-1 care. *PLoS One*.

